# Sleep and β-Amyloid Deposition in Alzheimer Disease: Insights on Mechanisms and Possible Innovative Treatments

**DOI:** 10.3389/fphar.2019.00695

**Published:** 2019-06-20

**Authors:** Susanna Cordone, Ludovica Annarumma, Paolo Maria Rossini, Luigi De Gennaro

**Affiliations:** ^1^Department of Psychology, University of Rome “Sapienza,” Rome, Italy; ^2^Department of Neurological, Motor and Sensory Sciences, IRCCS San Raffaele Pisana, Rome, Italy; ^3^Institute of Neurology, Catholic University of The Sacred Heart, Rome, Italy

**Keywords:** sleep, β-amyloid, Alzheimer’s disease, glymphatic system, slow-wave activity

## Abstract

The growing interest in the preclinical stage of Alzheimer’s disease (AD) led investigators to identify modifiable risk and predictive factors useful to design early intervention strategies. The preclinical stage of AD is characterized by β-amyloid (Aβ) aggregation into amyloid plaques and tau phosphorylation and aggregation into neurofibrillary tangles. There is a consensus on the importance of sleep within this context: the bidirectional relationship between sleep and AD pathology is supported by growing evidence that proved that the occurrence of sleep changes starting from the preclinical stage of AD, many years before the onset of cognitive decline. Hence, we review the most recent studies on sleep disturbances related to Aβ and the effects of sleep deprivation on Aβ accumulation in animal and human models. We also discuss evidence on the role of sleep in clearing the brain of toxic metabolic by-products, with original findings of the clearance activity of the glymphatic system stimulated by sleep. Furthermore, starting from new recent advances about the relationship between slow-wave sleep (SWS) and Aβ burden, we review the results of recent electroencephalographic (EEG) studies in order to clarify the possible role of SWS component disruption as a novel mechanistic pathway through which Aβ pathology may contribute to cognitive decline and, conversely, the eventual useful role of SWS in facilitating Aβ clearance. Finally, we discuss some promising innovative, effective, low-risk, non-invasive interventions, although empirical evidence on the efficacy of sleep interventions in improving the course of AD is at the very beginning.

## Introduction

Alzheimer’s disease (AD) is the most common cause of dementia and represents one of the most dramatic challenges of modern society. The increase in elderly population and life expectations and the public health and economic challenges led investigators to develop sensitive biomarkers, risk and predictive factors able to facilitate early detection and effective intervention strategies (Sperling et al., 2011).

The relationship between sleep and AD is well known: a high percentage of AD patients complained sleep disturbances along the entire course of the disease, increasing in severity with the progression of AD (Prinz et al., 1982; Vitiello et al., 1990; Moe et al., 1995).

Assuming that AD pathophysiology occurs many years before the manifestation of cognitive decline, recent literature data show that research in neuroscience is focusing on the preclinical stage of AD. This stage is characterized by deposition of extracellular amyloid-β (Aβ) into insoluble plaques in the brain associated with the aggregation of protein tau into intracellular neurofibrillary tangles (e.g., Lucey and Bateman, 2014). Amyloid deposition can be measured *in vivo* in humans by cerebrospinal fluid (CSF) Aβ concentration levels and by positron emission tomography with the amyloid tracer Pittsburgh compound B (PET-PiB). Through the use of both these measures, it has been observed that amyloid deposition was present approximately in 25–30% of cognitively intact individuals in their eighth decade (Morris et al., 2009).

Starting from the assumption that the intervention strategies are most effective in the preclinical stage of the disease, new research lines are investigating the modifiable factors occurring during this stage, together with the neuropathological events. In this context, the role of sleep in relation with Aβ is increasing in importance: sleep disturbances are present since the occurrence of Aβ accumulation, denoting a strict bidirectional relationship between sleep and Aβ. The subjective and objective measures of fragmented sleep are associated with the degree of Aβ accumulation and Aβ levels in the CSF (Lim et al., 2013; Spira et al., 2013; Mander et al., 2015).

For both animal and human models, it has been observed that sleep deprivation (SD) causes the augmentation of soluble Aβ (Kang et al., 2009; Ooms et al., 2014). Furthermore, in the mouse model, SD provokes also an increase in amyloid plaque deposition (Roh et al., 2014).

With the aim of investigating which aspects of sleep could be responsible for modulation of Aβ, an increasing number of electroencephalographic (EEG) studies (e.g., Ju et al., 2017) explored the role of slow-wave sleep (SWS) and specific non rapid eye movements (NREM) SWS components—with particular reference to slow-wave activity (SWA)—as candidates in the clearance of Aβ, promoting glymphatic system activity. The glymphatic system is a perivascular network diffused in the brain that has the role of achieving the exchange in interstitial fluid and CSF (Boespflug and Iliff, 2018) and is mainly implied in the clearing process of Aβ and other interstitial solutes. Growing evidence demonstrated that the glymphatic system mainly is active during sleep and impaired with aging and post-traumatic brain (Iliff et al., 2014; Kress et al., 2014; Zeppenfeld, 2017). 

Here, we briefly describe the bidirectional relationship between sleep and Aβ. Then, we review recent literature with particular reference to the last decade, reporting empirical evidence on the relationship between sleep disturbances and Aβ in elderly populations, and the most recent SD experimental data in animal and human models. We also discuss a new concept “beyond amyloid,” underlying the importance of other factors—related directly and indirectly to sleep—that has been receiving growing interest as contributors in the pathophysiology and progression of AD. On the basis of the most recent advances, we also discuss findings on the role of sleep in clearing the brain of toxic metabolic by-products, providing the results of new studies about the clearance activity of the glymphatic system stimulated by sleep, with particular reference on the role of SWA.

On the basis of the reviewed data, we also report a recent promising research line, describing innovative early sleep intervention strategies.

## Sleep and Aβ: A Bidirectional Relationship

For over 25 years, sleep disorders have been associated with AD, with a 25–66% of AD patients that exhibit sleep disturbances being considered one of the leading causes of patient institutionalization (Moran et al., 2005; Guarnieri et al., 2012).

During the last years, with the growing interest in the preclinical stage of AD, the role of sleep in association with AD has radically changed. Sleep changes occur many years before the appearance of cognitive symptoms, together with the early pathophysiological events. The presence of sleep disturbances, since the preclinical stage of the disease, underlines a possible crucial role of sleep in AD pathology and progression.

In 2009, Kang and colleagues showed in the AD mouse model that Aβ levels in the interstitial fluid increased during wakefulness and decreased during sleep. After this pioneering finding, many observational studies investigated on poor sleep as a potential human AD biomarker. Two physiological mechanisms could explain how poor sleep could promote AD: i) during SWS, the brain could be able to better clean metabolic waste, and Aβ clearance would be more effective during SWS (Xie et al., 2013); and ii) another mechanism was based on evidence that increased neuronal firing could promote Aβ production, and the firing is reduced in SWS as compared with wakefulness or REM sleep, and, consequently, sleep loss could lead to increase neuronal activity resulting in Aβ increase (Vyazovskiy et al., 2009; Ju et al., 2014). At this purpose, it is important to underline that literature data remain controversial. In particular, the open issue seems to be related to the different frequencies of neuronal firing: it is known that the majority of cortical neurons fire at low frequency (Chauvette et al., 2010; Barth and Poulet, 2012). Starting from this assumption, while sleep seems to reduce the firing of high-firing-rate neurons, in case of very-low-frequency-firing (around 1 Hz) neurons, it seems to augment the firing as underlined in evidence conducted in both rodents and cats (Watson et al., 2016). Moreover, Grosmark et al., (2012) examined the firing rates of hippocampal CA1 neurons and found that only REM episodes were related to a decreased firing rates at hippocampal level, revealing also an increase in firing during NREM sleep episodes.

During the last 15 years, many studies assessed the relations between subjective and objective sleep measures and increasing Aβ levels and lower cognitive performance in elderly populations, suggesting that poor sleep could increase the risk of obtaining low cognitive outcomes (Scullin and Bliwise, 2015). The focus on healthy elderly population represents a crucial methodological advance from earlier studies conducted in already-impaired older adults (Spira and Gottesman, 2017). Indeed, in the light of developing the most effective and early interventions, it becomes necessary to evaluate a series of age-related characteristics in a healthy population and, ideally, following across time any change in different physiological, structural, functional, and behavioral aspects.

Hence, current research lines are focusing their attention on the relationship between Aβ, sleep, and cognitive function in different experimental conditions, with particular reference to healthy elderly populations by investigating sleep disturbances and sleep deprivation related to Aβ burden.

## Aβ and Sleep Disturbances: A New Perspective

Growing evidence supports the notion that insomnia, excessive daytime sleepiness (EDS), sleep-disordered breathing (SDB), and circadian sleep–wake alterations all seem to increase the risk of AD (for a review, see Yaffe et al., 2014). It has been shown that sleep disorders modify neurotransmitter activity that could cause consequent dysfunction of the “default mode network,” which has a crucial role in the pathophysiology of AD (Yulug et al., 2017).

Following the new perspective of Aβ and sleep bidirectional relationship, many studies investigated whether sleep disruption could lead to deleterious effects on Aβ accumulation in healthy populations (e.g., Cross et al., 2015).

Recently, Chen and colleagues (2018) assessed the CSF Aβ levels in 23 patients with chronic insomnia, to reveal the potential effects of chronic sleep lack on the pathogenesis of AD. The authors found that CSF Aβ42 levels are significantly increased in insomniac patients. Furthermore, Aβ levels significantly correlated with the Pittsburgh Sleep Quality Index (PSQI) scores (i.e., the most used self-report measure that assesses sleep quality).

Carvalho and co-workers (2018) conducted a longitudinal study to assess the association between EDS and Aβ levels, through the use of PiB-PET scans across time (3 years’ follow-up). The results show that cognitive normal elderly (*n* = 283) with a high score of EDS at baseline condition were subjected to a higher risk of developing changes related to AD, as shown by the progressive increase of their Aβ levels over 3 years. 

A similar longitudinal experimental design has been applied by Sharma and colleagues (2017) to investigate the association between obstructive sleep apnea (OSA) and Aβ levels in a cognitively intact elderly population (*n* = 208). The results show that high OSA indexes were related to higher Aβ levels measured by PiB-PET scans and the increasing in Aβ levels progressively augmented across time (2 years’ follow-up).

The strong limitation of these studies is, however, that their assessment of sleep disturbances was based only on a self-report questionnaire, without the use of objective or clinical measures.

Some of these observations were, however, confirmed in studies conducted using actigraphy to examine sleep disorders related to preclinical AD. For example, Lim and co-workers (2013), through a prospective actigraphic study with 10 consecutive sleep recordings, show that participants with higher sleep fragmentation have a risk of developing AD symptoms in the successive 3 years, which is 1.5 times higher if compared with that of participants having lower fragmented sleep.

It is interesting to note that sleep fragmentation characterized, directly or indirectly, all these sleep disturbances: insomnia, EDS, and OSA are representative of poor sleep quality, restricted duration, and difficulty in maintaining sleep continuity.

The need for exploring sleep disturbances associated with Aβ with objective measures seems mandatory at this stage of evidence. Objective measures should include sleep EEG recordings, to obtain important details concerning macro-structural and micro-structural EEG measures. 

Particular attention should be given to the changes in the sleep–wake cycle. Aging *per se* leads to many changes at the physiological level, and these modifications concern also sleep and circadian rhythm and could derive from hypothalamic functional alterations (Monk et al., 2011). Indeed, lateral hypothalamus contains also neurons that impact wakefulness, in terms of initiating and maintaining wakefulness state (Chemelli et al., 1999). This role seems to be accomplished by orexin (hypocretin) neuropeptides that could be considered as a sort of substrate connecting dysfunctional homeostatic and cognitive processes in case of aging or AD and has been receiving growing attention also concerning new intervention strategies based on sleep restoration (Guarnieri et al., 2014).

The hypothesis of a relevant role of orexin neuropeptide in AD has also been confirmed by a study conducted with transgenic 2567 (TG2567) mice. This type of mouse model is commonly used in AD experimental procedures because it presents a mutation of the amyloid precursor protein but does not contract signs of AD at the behavioral level. Results show that orexin intracerebroventricular administration during inactive periods (that probably correspond to sleep) lead to an augmentation of wakefulness periods and Aβ levels in interstitial fluid (Kang et al., 2009).

Many evidence shows that alterations in circadian rhythm and sleep–wake cycle are strictly related to Aβ pathology: high Aβ levels are associated with fluctuations in alertness (e.g., Musiek et al., 2015), which contribute to the successive occurrence of “sundowning” with progressive neurodegeneration across time.

Considering animal models, the major concentrations of Aβ occur during wakefulness in TG2567 mice and wild type (WT; Kang et al., 2009), and, similarly, human models denote significant differences in Aβ levels measured during wakefulness (maximum concentration) and during normal sleep (minimum concentration; Huang et al., 2012).

The changes concerning sleep–wake cycle alterations include nocturnal sleep fragmentation, increased wakefulness, and functional impairment in daytime activity with diurnal napping (Vitiello and Prinz, 1989; van Someren et al., 1996). The specific sleep alterations in the preclinical stage of AD regard SWS, while REM sleep seems to be affected in later stages, with the progression of the disease (Vitiello and Prinz, 1989). Although brain remains electrically and metabolically active during sleep, a reduction of functional connectivity occurs at sleep onset (Vecchio et al., 2017) and with increasing depth of NREM sleep, with the maximal reduction observed during stage 3 (N3) of SWS (Horovitz et al., 2009). It has been hypothesized that the decrease during N3 could be due to decreased neuronal activity in this sleep stage. In support of these findings in humans, Fernandez and colleagues in a study conducted on mice found similar results regarding the decreased connectivity during sleep (Fernandez et al., 2017).

A recent interesting contribution investigated the impact of Aβ on the sleep–wake cycle, demonstrating that Aβ disturbs the synchronization of the central nervous system clocks, which are crucial for many processes. The worsening in synchronization is typical of normal aging and could further worsen, leading to neurodegeneration (Cedernaes et al., 2017).

This hypothesis is also supported by 24-h fluctuations in CSF Aβ levels measured by PET that decrease with age in “amyloid positive” healthy adults (Kang et al., 2009; Huang et al., 2012).

## The Effects of Sleep Deprivation on the β-Amyloid Accumulation

SD studies have been providing crucial advancements in the understanding of the mechanisms underlying the intriguing bidirectional relationship between sleep and Aβ.

It is well known that, in general, SD is strictly associated with cognitive impairment. Both clinical and experimental studies show that sleep loss, even for a few hours, provokes cognitive impairments. A wide range of empirical evidence demonstrates impairments in memory, learning, attention, decision making, and emotional reactivity in healthy human subjects after sleep loss (Chee and Chuah, 2008; Goel et al., 2009; McCoy and Strecker, 2011).

SD experimental contributions derive from both animal and human models, and although animal models represent a fundamental approach in the field of translational research in AD, the findings on the animal model do not completely overlap those in humans.

Studies manipulating SD make a distinction between total sleep deprivation (lack of sleep for a specific period extending to a longer period of sleep), mild sleep restriction (long-term shortening of the usual duration of sleep), and sleep fragmentation (interrupted sleep in different stages).

Starting from the assumption that sleep loss has an important negative implication in cognitive impairment, the most recent contributions of animal and human models in the field of AD are extending their focus on other interesting methodological and conceptual issues: i) scheduling different long-term SD periods; ii) investigating different subtypes of cognitive impairments; iii) testing the irreversibility of these impairments, by utilizing longitudinal experimental protocols; iv) increasing the use of non-genetically predisposed mouse model to better replicate adult-elderly individuals; v) considering sleep loss as a “stressor”; vi) investigating possible neuronal mechanisms implied in sleep–Aβ relationship; and vii) considering the possible role of glia in AD pathogenic mechanisms.

### Animal Models

Firstly, Rothman and colleagues (2013) tested the hypothesis that long-term mild restriction could worsen AD progression using the TG model with plaques and tangles, the 3×TgAD mouse model (*n* = 10). Mice were subjected to 6 h of sleep restriction per day for 6 weeks. The results showed that after chronic sleep restriction, there was an accentuation of Aβ accumulation in the cerebral cortex, demonstrating for the first time that long-term mild sleep restriction could worsen AD progression. Furthermore, behavioral data also reveal a worsening of memory loss in sleep-restricted mice if compared with controls. This study also analyzed corticosterone circulating levels, which were elevated 1 week after the start of the SD period and lasted for 4 weeks. The increase in corticosterone levels led to consider SD also in relation to stress intrinsically inherent to the procedures to sleep deprive, indicating that mice could not be able to acclimate to a stressor.

The notion of SD acting as a potential stressor was confirmed in other mice model studies. In particular, a successive study by Di Meco and co-workers (2014), utilizing the same 3×TgAD mouse model (*n* = 18), evaluated the functional and biological consequences of 4-h SD per day for 8 weeks. Compared with controls, mice subjected to SD show impaired cognitive performance in learning and memory abilities, but there are no significant differences in Aβ accumulation.

Interestingly, SD has an impact also in reducing postsynaptic density protein 95 levels with parallel augmentation of glial fibrillary acidic protein levels. This result confirms the importance of SD as a chronic stressor, able to influence also cognitive functions AD neuropathology.

Aiming to investigate the effects of chronic SD (mice underwent to 2-month SD; they were awakened from 12:00 PM to 8:00 AM of the next day) on cognition and Aβ accumulation in mice, Qiu and colleagues (2016) used a model of familial AD transgenic mice (*n* = 40) and their WT (*n* = 40). Results showed a worsening in cognition (learning and memory) in mice subjected to SD if compared with non-SD mice for both TG and WT models. Furthermore, the augmentation in Aβ accumulation and typical senile plaques was observed after SD in the cortex and hippocampus. It is interesting to note that the effects related to SD lasted for 3 months and remained stable also in normal, non-experimental conditions.

These findings underline that chronic SD could be considered as a potential risk factor for AD.

This is the first study demonstrating that reversal learning ability is defected by chronic SD. The authors assume that SD as a stressor can cause damage to both the hippocampus and cortex and it could aggravate dementia. This study explores the effects of chronic SD on both familial AD model and sporadic one, to facilitate the understanding of the association between chronic SD and AD. On the basis of those results, it is possible to suggest that chronic SD is not only a risk factor for familial AD but also contributes to sporadic AD. 

Familial (i.e., early-onset AD) AD typically accounts for a quite small percentage of all AD cases (e.g., Bali et al., 2012), and it becomes fundamental to evaluate whether chronic SD could facilitate Aβ accumulation and make vulnerable the non-genetically predisposed mice to the risk of dementia, specifically investigating sporadic AD (i.e., late-onset AD, which is the most common AD form). In a recent study of Zhao and co-workers (2017), non-genetically predisposed mice (adult and WT C57BL/6 mice) were submitted to chronic SD for 2 months (equivalent to 6–8 years in humans). Mice usually sleep 10–12 h each day, and experimental SD protocol allowed mice to sleep restriction (4 h per day). This study had the aim of verifying alterations in cognitive function and Aβ levels after a period of SD. Results showed that cognitive function worsened for both learning and memory domains. Concerning Aβ accumulation, the most affected brain areas were at the prefrontal and temporal lobe levels, differing from above-mentioned findings, suggesting that it could be possibly due to variant genetic background.

Beyond the traditional mice models, the fruit fly *Drosophila melanogaster* has been established as a model for AD (e.g., Bonner and Boulianne, 2011) because it recapitulates some key pathophysiological and cognitive characteristics of AD (Tabuchi et al., 2015). A recent study of Tabuchi and colleagues (2015) hypothesized a functional mutual interaction that includes sleep, neuronal excitability, and Aβ levels also in animals different from mice model. To this purpose, using mechanical deprivation, authors subjected flies to SD for 1 week, starting from the assumption that sleep functions downscale synaptic strength and SD could have a deleterious effect on neuronal excitability related to AD pathology. The results of this study underline an augmentation in neuronal excitability due to abundant Aβ accumulation and a worsening of this effect after SD. These changes in electrical activity are also linked to cognitive impairment: mild cognitive impairment (MCI) patients showed augmented hippocampal activation by functional magnetic resonance imaging (fMRI), while its reduction improved memory performance (Bakker et al., 2012). The findings of this study raise the important issue of the strong interaction between sleep and Aβ, adding the negative relevance of neuronal hyperexcitation in case of sleep loss and, on the contrary, suggesting a possible beneficial role of intact sleep in AD pathophysiology.

### Human Models

The first human study by Ooms and colleagues (2014) investigated the effects of one night of total SD on CSF Aβ42 levels in healthy middle-aged men (*n* = 26). A night of undisturbed sleep led to a 6% decrease in Aβ42 levels, whereas this decrease was not observed in case of sleep restriction. Furthermore, Aβ42 levels in a different time of the day were significantly different between the two groups in contrast to Aβ40 and tau protein levels that remained stable. These results suggest that SD could interfere with the physiological decrement in Aβ42 levels measured in the morning, and it elevates the risk of AD. Another study (Wei et al., 2017) tested the effects of short-term total sleep deprivation on plasma Aβ levels. Twenty healthy volunteers underwent 24 h of SD. Results show that SD can provoke Aβ40 plasma level augmentation and a decrement in Aβ42/Aβ40 ratio. As mentioned above for animal models, these results underline a possible mechanism that also implies oxidative stress in the impairment of peripheral Aβ clearance as a pathophysiological mechanism of AD. The plasma Aβ40 level was linearly correlated with the time of wakefulness, and the changes were reversed after sleep recovery. These sleep-related effects were not observed for plasma Aβ42.

In contrast, Lucey and colleagues (2017) found that disrupted sleep increases AD risk *via* increased Aβ38, Aβ40, and Aβ42 levels by 25% to 30% in cognitively normal adults (*n* = 8; aged 30 to 60 years).

Using PET, Shokri-Kojori and colleagues (2018) showed that acute SD impacts the Aβ burden in brain regions implicated in AD. In particular, the authors evaluated the effects of one-night SD on Aβ burden in healthy controls (*n* = 22). The increase in Aβ burden was found in the hippocampus and thalamus, confirming that SD has an impact on brain regions strictly connected with AD pathology starting from the first stages of the disease.

The most recent studies are trying to increase in the extent of SD manipulation. Olsson and co-workers (2018) investigated the effect of five consecutive nights of partial sleep deprivation (PSD) on CSF biomarkers in healthy adults (*n* = 13). After baseline condition (8 h per night for five nights), PSD protocol consisted in reducing sleep to 4 h for the successive eight nights. CSF biomarker included Aβ, tau, orexin, monoamine metabolites, neuron-derived biomarkers, and astro- and microglia-derived biomarkers. Results showed that five to eight consecutive nights of PSD only affected CSF orexin levels, in terms of augmentation. No impact was found on the other CSF biomarkers.

The above-mentioned empirical evidence related to animal and human models, with particular reference to new interesting methodological and conceptual issues, has provided new important insights going towards a “beyond amyloid” concept of AD pathologic mechanisms.

## AD as a Multifactorial Disease: Beyond Amyloid

The growing evidence on AD pathogenesis is depicting a new conceptual and experimental framework that overcomes the mere “amyloid cascade hypothesis,” leading to even more complex considerations on other factors that could contribute, reciprocally interact, and have a role in AD pathogenesis and progression. Clinicians and researchers are increasingly considering AD as a multifactorial disease syndrome, trying to identify the roles of these different factors in relation to AD pathogenesis and progression. In recent literature, an open issue concerns the importance and the weight of the different “contributors” and their mutual interactions. A recent commentary of Mullane and Williams (2018) underlines the new consideration of AD as a “complex cellular phase consisting of feedback and feedforward responses of astrocytes, microglia, and vasculature” and a “holistic understanding of the spatial, temporal and cellular aspects of the disease process” (De Strooper and Karran, 2016) is required. In their commentary, authors did not question the already-known notions about Aβ burden as a key pathological feature in AD, but they suggest a complex framework that goes “beyond amyloid” in the understanding of AD pathogenesis. This concept also depends by clinical evidence that shows substantial independence of neurodegenerative signs (such as Aβ accumulation) from cognitive status: it has been shown that the removal of Aβ from AD brain could not have positive effects on cognition. This consideration also suggests that the amyloid cascade hypothesis of AD could be rejected (Egan et al., 2018).

In the perspective of AD as a multifactorial disease, recent studies present many causal factors implicated in AD. These factors include inflammation (McGeer et al., 1990), neurotoxic protein accumulation in the brain (Boland et al., 2018) that can be associated in part with sleep deprivation, disruption of the glymphatic system and blood–brain barrier (BBB) dysfunction (Jessen et al., 2015; Sweeney and Zlokovic, 2018), oxidative stress, and microglia dysfunction. None of these factors could provoke, independently, AD occurrence, but their combinations and interactions could have a role in facilitating its occurrence. The role of these contributing factors could clarify the fact that a high percentage of individuals that present signs of AD, such as amyloid plaques, do not show impairment in cognition (Aizenstein et al., 2008); and, on the contrary, it is possible to develop AD without observed amyloid signs in the brain (Nelson et al., 2011; Herrup, 2015).

Within this new framework, the most relevant issue concerns how sleep is inserting in the mechanistic pathways underlying AD pathogenesis, to develop new preventive and early intervention strategies.

It is important to underline that many recent contributions investigated sleep also in relation to tau protein with dissociated findings for changes of Aβ and tau (e.g., Holth et al., 2017). Also, for this reason, the relationship between sleep and tau is not the subject of the present review.

## The Role of Sleep in Clearing the Brain of Toxic Metabolic By-Products

Recent reports indicate a strict relation between disrupted sleep, brain glymphatic system, and AD (O’Donnel et al., 2015; Krueger et al., 2016). Removing waste from the central nervous system is crucial for the maintenance of brain homeostasis during life span, and, at this purpose, the role of the glymphatic system has been profoundly investigated. The glymphatic system includes a perivascular network for CSF transport (Iliff et al., 2012) connected to a downstream lymphatic system (Aspelund et al., 2015; Louveau et al., 2015). Xie and colleagues (2013) conducted the first pioneering studies that showed as Aβ protein was transported from the interstitial fluid space and out of the brain through the glymphatic system. The association between sleep and glymphatic system derived from evidence that demonstrate as sleep (with particular reference to SWS) augmented the action of the glymphatic system in clearing Aβ from the brain if compared with wakefulness state, suggesting a possible beneficial role of sleep also in intervention strategies with the aim of ameliorate cognitive status (Benveniste, 2018). The role of SWS in the clearance of Aβ *via* glymphatic system was tested by Xie and co-workers in an animal model: Aβ in rodent brain was cleared significantly faster in the cortex during SWS if compared with wakefulness. Although the presence of a glymphatic system in the human brain has not been proven, several correlation studies based on the investigation of the relationship between Aβ levels in the CSF and sleep/wake variables—together with SD deprivation evidence—hypothesized a plausible presence of a glymphatic system also in the human brain (Volkow et al., 2012; Xie et al., 2013). Furthermore, other evidence refers, specifically, to i) SWS disruption with a parallel increase in CSF Aβ levels (Ju et al., 2017) and ii) correlation of subjective measures regarding sleep duration and Aβ levels in the brain (Spira et al., 2013).

Again, the importance of sleep in Aβ clearance has also been confirmed in relation with a significant increase in interstitial fluid space volume at the cortex level in sleeping rodents than in wakefulness periods (Xie et al., 2013; Ding et al., 2016).

Animal and human models showed that Aβ levels in the interstitial fluid undergo diurnal oscillations (Musiek et al., 2015) that seem to be due to decreased neural activity in SWS during NREM sleep in some brain areas, which could be linked to a decrement in Aβ production. Considering the specificity of SWS in clearing Aβ, a series of new experimental protocols have been conducted to clarify the role of SWS on the light of the new findings mentioned above.

## The Relation Between Slow-Wave Activity (Swa), Nrem Oscillation Components, β-Amyloid Burden, and Brain Structural and Functional Differences

By reviewing recent literature on sleep and AD relationship, it is evident that most experimental contributions have been conducted in healthy samples, in line with the concept of investigating the earliest preclinical pathological events related to AD and the possibility to target preventive and early-based sleep intervention.

Furthermore, the most recent innovative framework in the field of sleep and AD relationship derives from another new methodological perspective. The most important findings of the last decade show as the combination of different measurements represent the best overview to provide a complete vision of anatomical, electrophysiological, metabolic, and behavioral aspects underlying this relationship (**Supplementary Table 1**). In the light of SD animal and human models mentioned above, it emerges the need for examining specific brain regions—related to sleep—involved in AD pathogenesis. It becomes essential to investigate the complex interactions among the features related to AD with particular reference to the mechanistic pathways that could link specific Aβ accumulation in the brain, sleep state, and cognitive impairment (memory, at first).

Concerning the electrophysiological measurement of brain activity, the investigation of changes in electrical oscillations of sleep through EEG, with particular reference to NREM constituent oscillations—such as slow oscillations and sleep spindles—acquires great importance in this research line.

In particular, one quantification of slow waves entails the measurement of spectral EEG power in the 0.5- to 4.5-Hz range during SWS, also known as SWA. Studies conducted in healthy populations showed significant SWA power reductions with aging progression (Dijk et al., 1989; Landolt et al., 1996; Mander et al., 2013). In particular, the highest decreases in SWA are observed within the prefrontal cortex (PFC) during the first part of the night, in correspondence with the first NREM cycle (Landolt et al., 1996; Mander et al., 2013).

Slow wave is strictly related to gray matter within specific PFC regions: atrophy in these brain areas seems to predict the degree of changes in NREM slow-wave characteristics in elderly populations (Mander et al., 2013; Varga et al., 2016).

At this purpose, a relevant contribution of Mander and co-workers (2013) that combined MRI, fMRI, EEG, and memory measurements demonstrated that atrophy in the prefrontal gray matter predicted NREM SWA disruption, and the interaction between those measures predicted cognitive performance in overnight episodic memory retention. fMRI scans obtained during memory task showed that memory impairment was related to continuous hippocampal activation and reduced connectivity between the hippocampus and prefrontal cortex (Mander et al., 2013). 

Considering the importance of the early Aβ accumulation in the brain, SWA has been investigated in relation to Aβ levels in CSF and memory consolidation. It is well known that Aβ’s earliest occurrence appears at the cortical level and also includes the medial Prefrontal Cortex (mPFC) (Buckner et al., 2005). Indeed, subcortical structures are affected successively. For this reason, Mander et al. (2015) hypothesized involving the hippocampus since the preclinical stage of AD (clearly observed with early memory impairment) with regards the indirect pathways in which Aβ could impact the hippocampal–neocortical functioning. The hypothesized pathway is a sort of loop that includes NREM sleep disruption, SWA, and memory destruction (Mander et al., 2013).

The same research group conducted a successive study (Mander et al., 2015) on older adult population (*n* = 26), to clarify whether the extent of Aβ accumulation at mPFC level was related to decreased NREM SWA. Furthermore, the authors had the aim of proving whether NREM SWA correlated with the degree of memory impairment. Memory evaluation referred, specifically, to overnight hippocampus-dependent memory consolidation. Experimental protocol combined PET-PiB scanning to measure regional Aβ burden for one night of sleep EEG recording of behavioral and functional fMRI for sleep-dependent memory consolidation. This elegant study is the first evidence to demonstrate the correlation between cortical Aβ and impaired generation of NREM SWS, also related to the prediction of successive decline in the hippocampal–neocortical memory transformation and related overnight memory retention. The results of this study extend prior studies that showed the association among the accumulation of Aβ within mPFC, the NREM SWA impairment, and the further correlation of disrupted NREM SWA with impaired hippocampal–neocortical memory transformation and related overnight memory retention. Taken together, these results provide important insights at anatomical and electrophysiological levels, as discussed by the authors (Mander et al., 2015). Anatomically, through the analyses of source localization, a correspondence of mPFC regions impacted early by Aβ burden and slow-wave generator has been observed in healthy young adults (Sepulcre et al., 2013; Mander et al., 2015). At the electrophysiological level, an important distinction within the delta frequency range (1–4 Hz) emerges: the relation between Aβ and NREM SWA regards only the low-frequency range of SWA (0.6–1 Hz). So it suggests that only this specific frequency range is associated with Aβ pathology.

Considering microstructural features of NREM sleep, both amplitude and density of slow waves are significantly reduced across aging (Dubè et al., 2015), showing the largest changes over the frontal lobe and be maximal during the greatest expression of NREM oscillation, in the first one to two NREM cycles. These changes in the morphology of waveforms could be due to the diminishing of synchronized neuronal firing that causes sleep oscillations, provoked by a disruption in the slow wave depolarized or hyperpolarized down states that shape the slow wave (Beenhakker and Huguenard, 2009; Mander et al., 2017). Within the same frequency range of slow waves, AD patients compared with healthy controls show a 40% decrease of frontal spontaneous K complex during NREM sleep, and this decrease was positively related with Mini Mental State Examination (MMSE) scores (De Gennaro et al., 2017). The lack of a similar decrease in the amount of SWA suggests that the <1-Hz slow activity could be the potential sleep EEG marker, even though it does not differentiate AD from MCI patients (Reda et al., 2017).

Concerning sleep spindles, these NREM characteristic features reflect transient bursts of oscillatory activity in the 12- to 15-Hz range and are generated through an interaction between cortico-thalamic networks and reticular nucleus of the thalamus (De Gennaro and Ferrara, 2003). Rauchs and colleagues (2008), for the first time, reported a specific decrease in spindle in AD patients in association with learning abilities, underlying that these changes involved only fast spindles (13–15 Hz). Other studies demonstrated that the spectral power in this frequency range is decreased in middle-aged and older adults relative to young adults and is maximal over frontal EEG derivations (De Gennaro and Ferrara, 2003; Mander et al., 2014). A decreased rate of sleep spindles in older adults is also negatively associated with structural brain integrity: empirical evidence points to subcortical reductions in the gray matter within the hippocampus, and this decrement seems to predict the extent of decreased sleep spindle density at frontal lobe level in older adults (Fogel et al., 2017). In 2012, Wersterberg and colleagues investigated sleep physiology and memory in amnestic MCI patients (aMCI; *n* = 10) during stage 2 (i.e., the sleep stage where spindle activity is maximally expressed). The results showed that aMCI patients had fewer stage 2 spindles than aged-matched healthy adults (*n* = 18).

Furthermore, aMCI patients, if compared with controls, show less time spent in SWS and lower delta and theta power. Importantly, sleep deficiency in aMCI was also implicated in declarative memory consolidation: altered sleep patterns seem to contribute to memory impairments by contrasting with sleep-dependent memory consolidation. In this study, reduced stage 2 spindles regarded fast (typically 13–15 Hz) but not slow spindles, in line with previous contributions that showed that fast spindles are most disrupted in AD (Rauchs et al., 2008), and these reductions were observed at frontal recording sites (Bliwise, 1993). A decrease of parietal fast spindle density in AD and MCI patients compared with healthy controls was confirmed by others (Gorgoni et al., 2016). This decrease positively correlated with MMSE scores.

The role of NREM activity, including the expression of sleep spindle EEG oscillations, has been further investigated by Mander and colleagues (2014). The authors started from the hypothesis that sleep could have a role in improving learning ability, in terms of better restoring next-day encoding learning abilities (e.g., Yoo et al., 2007). This role could be due, specifically, to the activity of fast-frequency sleep spindles over the left prefrontal cortex, as previously observed in healthy young adults (Mander et al., 2011). Experimental protocol combined fMRI and EEG recordings to i) evaluate the role of prefrontal fast sleep spindles in predicting next-day episodic encoding ability and ii) assess whether disruption in this EEG activity decreases the effectiveness of this positive benefit on hippocampal-dependent episodic learning ability.

The results showed that decreased prefrontal sleep spindle in older adults statistically mediated the effect of old age on next-day episodic learning and that the extent of this impairment in learning ability seems to depend on the degree of decreased prefrontal sleep spindles. Prefrontal spindle activity also seems to be related to hippocampal activation and could have an impact on learning ability in post-sleep phase. These results contribute to confirming the hypothesis that disrupted sleep physiology has a role in the age-related cognitive decline in later life (Mander et al., 2014).

It is interesting to note that there is a strong resemblance concerning source generators of NREM SWS oscillations, which preponderate at mPFC level, and brain areas in which Aβ mainly accumulate in both cognitively healthy older adults and AD patients (e.g., Murphy et al., 2009).

NREM SWS disruption is exacerbated early in the course of AD, and this decrease predicts the severity of observed memory impairment (Wersterberg et al., 2012; Liguori et al., 2014). Finally, recent studies in human and rodents demonstrate that interstitial Aβ levels rise and fall with the brain states of wake and NREM sleep, respectively: shorter NREM duration and greater NREM fragmentation have been reported in mice over-expressing Aβ proteins (Kang et al., 2009; Roh et al., 2012), while human subjective reports of reduced sleep duration and diminished sleep quality correlate with cortical Aβ burden in healthy older adults (Spira et al., 2013). Moreover, direct manipulations of sleep and Aβ production in rodent models of AD have established bidirectional relationships between both factors (Kang et al., 2009; Roh et al., 2012; Xie et al., 2013).

The role of disrupted NREM sleep in contributing to the impairment of new hippocampal-based memory has also been evaluated through an interesting experimental method. Evidence shows that disturbing deep NREM sleep by applying acoustic stimulation during SWS coupled with spindle activity [when clicks are presented in synchrony with upcoming slow oscillation (SO) up states] had an impact on next-day cognitive performance (Ngo et al., 2013).

The experimentally induced increase in NREM SWA (in particular in the slow <1-Hz frequency range) seems causally enhancing subsequent consolidation and, thus, long-term memory retention in young adults (Marshall et al., 2006). In sleeping humans, Ngo and colleagues (2013) applied auditory stimulation in the phase with the ongoing slow oscillatory EEG activity, in order to enhance train of slow-wave oscillation (SO; <1 Hz) and assess next-day memory performance. Results show that declarative memory improved after stimulation, but only after in-phase stimulation with the ongoing slow oscillation rhythm (Ngo et al., 2013), underlying the possible role of SO in getting better memory performance.

These findings suggest that the effectiveness of acoustic stimulation in enhancing SO and improving memory performance could be mainly due to the timing in phase with slow oscillatory activity, as shown also in transcranial direct current stimulation: the experimental augmentation of SO intensity improved cognitive abilities, in terms of better performance in post-sleep hippocampal-dependent learning capacity (Antonenko et al., 2013).

To summarize, the role of SWS NREM components discussed above could inspire new experimental intervention approaches, based on non-invasive, low-cost, and effective preventive strategies.

## Possible Innovative Treatments

The importance of developing sleep preventive and intervention strategies derives from the assumption that sleep could be considered as a modifiable and treatable risk factor for AD.

Some behavioral practices are well known and of concern, at first, due to the use of sleep hygiene in AD. In general, sleep hygiene guidelines suggest some preventive behaviors in terms of i) limiting the use of psychoactive substances (e.g., caffeine, alcohol, or smoking); ii) avoiding light exposure from television or computer in the evening; iii) practicing regular physical exercise; and iv) keeping constant bed and wake times with adequate light exposure upon waking (e.g., McCurry et al., 2012).

Some evidence suggests that physical and social activities could improve sleep quality, and the major benefits derived from multimodal treatments that combine sleep hygiene education, light physical exercise (walking), and bright light therapy have been observed (McCurry et al., 2005).

Concerning pharmacological treatment of sleep deficiency in AD, three drugs have been tested as alternatives to traditional hypnotics: melatonin, trazodone, and ramelteon (Mccleery et al., 2014), but the reported efficacy only concerned mild, moderate, and severe AD patients, without being proved in preclinical stages of AD.

In general, the effectiveness of melatonin is the most investigated. A recent review (Xu et al., 2015) evaluated the effectiveness of melatonin assumption for sleep disturbance and cognitive function in dementia patients observed in seven studies (*n* = 520). Results showed that the use of melatonin protracted total sleep time (TST) and, marginally, sleep efficiency, but cognitive function did not change significantly.

Going beyond the already known non-pharmacological strategies, in the light of the new findings on the role of SWS related to AD, it is possible to propose a possible intervention based on NREM SWS. In particular, Mander and colleagues (2016) suggest that NREM sleep enhancement in midlife to late life may lead to a preventive positive effect that could decrease AD risk, probably improving Aβ clearance or combating oxidative stress. Consequently, sleep restoration could also improve memory consolidation. Those authors suggest several promising tools for achieving NREM SWS enhancement benefit, with particular reference to <1-Hz NREM SWA. Currently, the experimental results associated with <1-Hz NREM SWA are contrasting: transcranial Direct Current Stimulation (tDCS) in the <1-Hz range seems to be effective in memory consolidation in young and older adults, in patients with schizophrenia, in individuals with attention-deficit hyperactivity disorders, and in patients with lobe epilepsy (Prehn-Kristensen et al., 2014; Del Felice et al., 2015). The results of other contributions show no benefits in memory consolidation in young and older adults (Eggert et al., 2013; Sahlem et al., 2015).

Enhancement of <1-Hz NREM SWA was also tested through auditory closed-loop stimulation during NREM SWS with promising positive outcomes on hippocampus-dependent memory consolidation (Ngo et al., 2013; Papalambros et al., 2017). Closed loop in phase auditory stimulation at low intensity might be considered a novel implement to improve restorative aspects of sleep rhythms, also in case of sleep disturbance (Riemann et al., 2011).

It is not possible to assume the real effectiveness of these methods, because, in our knowledge, there is no evidence of long-term benefits of this <1-Hz NREM SWA enhancement. 

## Concluding Remarks

This review recapitulates recent contributions on the relationship between sleep and Aβ. Evidence from the last decade, deriving from sleep disturbances and sleep deprivation in relation with Aβ, raises important conceptual and methodological issues in the field of AD research. The concept of AD as a multifactorial disease is growing up, and, consequently, the need for exploring different contributing factors in the pathogenesis and progression of the disease becomes fundamental in future perspectives.

The current findings on the relationship between sleep and Aβ have been providing important contributions i) to increase the understanding of the mechanisms underlined this relationship; ii) to understand the role of the different sleep stages in the pathogenesis and progression of AD; and iii) to target innovative, non-invasive intervention strategies based on sleep restoration.

Reviewing recent literature, the major contributions derive from studies that combine more measurements and foresee longitudinal experimental designs, extending their focus on many contributing factors, overlapping the mere amyloid cascade hypothesis. Currently, these studies are few, and further investigations are needed to confirm and extend the new promising finding in the field of sleep and AD.

## Author Contributions

SC wrote the manuscript with the contribution of LA in the bibliography search and in phase of writing. PR and LG have contributed and supervised to all the writing phases of the review. All authors agree to the final submitted version.

## Conflict of Interest Statement

The authors declare that the research was conducted in the absence of any commercial or financial relationships that could be construed as a potential conflict of interest.
